# Investigating gene signatures associated with immunity in colon adenocarcinoma to predict the immunotherapy effectiveness using NFM and WGCNA algorithms

**DOI:** 10.18632/aging.205763

**Published:** 2024-05-13

**Authors:** Weizheng Liang, Xiangyu Yang, Xiushen Li, Peng Wang, Zhenpeng Zhu, Shan Liu, Dandan Xu, Xuejun Zhi, Jun Xue

**Affiliations:** 1 Central Laboratory, The First Affiliated Hospital of Hebei North University, Zhangjiakou 075000, Hebei, China; 2 Department of General Surgery, The First Affiliated Hospital of Hebei North University, Zhangjiakou 075000, Hebei, China; 3 Department of Gastroenterology and Hepatology, The Second Affiliated Hospital of Chongqing Medical University, Yuzhong 400010, Chongqing, China; 4 Department of Obstetrics and Gynecology, Shenzhen University General Hospital, Shenzhen 518055, Guangdong, China; 5 Bioimaging Core of Shenzhen Bay Laboratory Shenzhen, Shenzhen 518132, Guangdong, China; 6 Department of Respiratory and Critical Care Medicine, The First Affiliated Hospital of Hebei North University, Zhangjiakou 075000, Hebei, China

**Keywords:** colon adenocarcinoma, immune microenvironment, WGCNA, immunotherapy, prognostic model

## Abstract

Colon adenocarcinoma (COAD), a frequently encountered and highly lethal malignancy of the digestive system, has been the focus of intensive research regarding its prognosis. The intricate immune microenvironment plays a pivotal role in the pathological progression of COAD; nevertheless, the underlying molecular mechanisms remain incompletely understood. This study aims to explore the immune gene expression patterns in COAD, construct a robust prognostic model, and delve into the molecular mechanisms and potential therapeutic targets for COAD liver metastasis, thereby providing critical support for individualized treatment strategies and prognostic evaluation. Initially, we curated a comprehensive dataset by screening 2600 immune-related genes (IRGs) from the ImmPort and InnateDB databases, successfully obtaining a rich data resource. Subsequently, the COAD patient cohort was classified using the non-negative matrix factorization (NMF) algorithm, enabling accurate categorization. Continuing on, utilizing the weighted gene co-expression network analysis (WGCNA) method, we analyzed the top 5000 genes with the smallest p-values among the differentially expressed genes (DEGs) between immune subtypes. Through this rigorous screening process, we identified the gene modules with the strongest correlation to the COAD subpopulation, and the intersection of genes in these modules with DEGs (COAD vs COAD vs Normal colon tissue) is referred to as Differentially Expressed Immune Genes Associated with COAD (DEIGRC). Employing diverse bioinformatics methodologies, we successfully developed a prognostic model (DPM) consisting of six genes derived from the DEIGRC, which was further validated across multiple independent datasets. Not only does this predictive model accurately forecast the prognosis of COAD patients, but it also provides valuable insights for formulating personalized treatment regimens. Within the constructed DPM, we observed a downregulation of CALB2 expression levels in COAD tissues, whereas NOXA1, KDF1, LARS2, GSR, and TIMP1 exhibited upregulated expression levels. These genes likely play indispensable roles in the initiation and progression of COAD and thus represent potential therapeutic targets for patient management. Furthermore, our investigation into the molecular mechanisms and therapeutic targets for COAD liver metastasis revealed associations with relevant processes such as fat digestion and absorption, cancer gene protein polysaccharides, and nitrogen metabolism. Consequently, genes including CAV1, ANXA1, CPS1, EDNRA, and GC emerge as promising candidates as therapeutic targets for COAD liver metastasis, thereby providing crucial insights for future clinical practices and drug development. In summary, this study uncovers the immune gene expression patterns in COAD, establishes a robust prognostic model, and elucidates the molecular mechanisms and potential therapeutic targets for COAD liver metastasis, thereby possessing significant theoretical and clinical implications. These findings are anticipated to offer substantial support for both the treatment and prognosis management of COAD patients.

## INTRODUCTION

Colon adenocarcinoma (COAD), one of the most common malignancies, is among the top five in terms of morbidity and death from tumor-related illnesses [1–4]. Predictions indicate that in 2022, colorectal cancer (CRC) diagnoses will number 600,000 in China and 160,000 in the US, with 300,000 and 55,000 cases of CRC-related deaths in each country, respectively [5]. The colon is anatomically more positioned inside than the rectum, which complicates diagnosis and therapy. The lack of accurate COAD indicators, which shows that most colon cancer patients have missed the opportunity for dramatic surgery by the time they are officially diagnosed, is one of the main reasons for the poor prognosis of COAD [6]. CRC is a disease that is well suited for screening since early detection of precancerous lesions greatly reduces the disease’s morbidity and death [7–10]. The overall survival of patients with COAD has not increased significantly despite significant breakthroughs in treatment [11]. Therefore, it is necessary to find prognostic biomarkers with high specificity or to create prognostic models with high predictive effect in order to oversee and guide the tailored treatment of COAD patients.

A variety of cytokines secreted during tumorigenesis and progression lead to the reprogramming of its surrounding stromal cells, which in turn promotes the proliferation and survival of tumor cells [12]. A significant factor in the formation, progression, and management of COAD is the interplay among immune cells, stromal cells, and cytokines within the tumor microenvironment [13]. In the tumor microenvironment, cytokines can be secreted by immune cells such as T cells, macrophages, and other immune cells, or produced by the tumor itself or stromal cells. These cytokines influence tumor growth, proliferation, and response to therapy by activating or inhibiting immune cells and controlling the tumor microenvironment [14, 15]. In addition to providing structural support, stromal cells, which include fibroblasts and vascular endothelial cells, control the recruitment and activation of immune cells through the secretion of cytokines [16]. In COAD, regulatory interactions between cytokines and immune cells may lead to the occurrence of immune escape, which promotes tumor cell proliferation and metastasis [17]. In the microenvironment of CRC, IL6 generated by CD163^+^ tumor-associated macrophages stimulates epithelial mesenchymal transition by controlling the STAT3/miR-506-3p/FoxQ1 pathway, which in turn promotes CRC cell invasion and migration [18]. Meanwhile, the accumulation of cytokine IL-6 can also promote the proliferation of CRC cells [19, 20]. Immunogenetic traits are associated with a better prognosis or greater effectiveness of immunotherapy for malignancies [21, 22]. Clinical research indicates that immune checkpoint inhibition combined with divalizumab and trimethoprim may increase overall survival in patients with advanced refractory CRC [23]. Not all colon cancer patients respond to immunotherapy [24]. The specific mechanism of action of the tumor microenvironment remains unclear, despite its major impact on immune efficacy [25, 26]. Therefore, it is an urgent requirement to screen for novel indicators to forecast the effectiveness and post-treatment response of immunotherapy to enhance the individualization of immunotherapy.

In this study, COAD samples were immune clustered using the non-negative matrix clustering (NMF) method based on genes relevant to immunity. Immunological clustering-related gene modules were found using the weighted correlation network analysis (WGCNA) algorithm. The intersection of the gene modules with the differentially expressed genes (DEGs) (COAD vs Normal colon tissue) was defined as differentially expressed immune genes related with COAD (DEIGRC). The DEIGRC prognosis model (DPM) was constructed using a variety of bioinformatics tools, and the expected accuracy of the DPM was confirmed using data from the Gene Expression Omnibus (GEO) database. The capability of the model to forecast treatment outcomes for cancer patients was evaluated, and the distinct immunological profiles among subgroups were characterized. The development of liver metastases in COAD patients was examined, along with possible processes and important therapeutic genes, using bioinformatics methods.

## MATERIALS AND METHODS

### Obtaining and processing IRGs and transcriptome sequencing data

The ImmPort database (https://www.immport.org/home) and InnateDB databases (https://www.innatedb.com/) provided the IRGs [27, 28]. Transcriptome sequencing data of 349 healthy colon and 471 COAD tissues were retrieved for this research work using UCSC Xena database (https://xena.ucsc.edu/). The “limma” package in R is used to obtain DEGs (|log (fold change)| > 1, *p*<0.05). The GEO database (https://www.ncbi.nlm.nih.gov/gds) was used to obtain the GSE17536, GSE39582, and GSE109211 datasets to verify prognostic models’ accuracy in predicting outcomes. The development and validation of prognostic models did not include samples that lacked clinical prognostic information. The GSE6988 dataset was used to explore putative biological processes that might underlie the growth of liver metastases in COAD patients.

### Immunophenotyping based on IRGs

We retrieved the expression data of IRGs from UCSC Xena database, which consisted of 471 samples from COAD patients. NMF analysis was performed on the screened data utilizing the ‘brunet’ criterion with the “NMF” package in R [29]. The ideal number of clusters was produced based on the results of the consensus clustering graph and residual sum of squares (rss), dispersion, and cophenetic graphs.

### WGCNA analysis

We used the “limma” package of R to compare the expression differences of genes between the immune subtypes, and then used the 5000 genes with the smallest *p*-value for WGCNA by ''WGCNA'' package in R [30]. The *p*-value of different genes between different immune subtypes was calculated through “limma” and 5000 genes with the smallest *p*-value were used for WGCNA. The steps were as follows: (1) In the data preprocessing stage, genes with standard deviation less than 0.5 were excluded to reduce noise interference and improve the reliability of the subsequent analysis. (2) By calculating the Pearson correlation coefficient between genes, the degree of linear correlation between genes was assessed, thus laying the foundation for the establishment of co-expression network. (3) Construct similarity matrix and neighboring matrix according to Pearson correlation coefficient to measure the correlation and connection strength between genes. (4) Determine the optimal soft threshold using the “sft” function in R, thus determining the topology and module division of the network. (5) Construct a topological overlap matrix (TOM) using the adjacency matrix and cluster genes into different modules. Clarify gene clusters with intrinsic relatedness by 1-TOM similarity transformation. (6) Evaluate the correlation between gene modules and immune subtypes, and obtain gene modules with strong correlation with immune subtypes. (7) Intersect genes from gene modules highly correlated with immune subtypes with DEGs (COAD with normal colon tissue) and define these intersected genes as DEIGRC.

### Bioinformatics analysis of DEIGRC

The “clusterProfiler” package and “enrichplot” package in R were employed to perform Gene Ontology (GO) and Kyoto Encyclopedia of Genes and Genomes (KEGG) enrichment analysis and enrich the potential relationships between the analysis results.

### Construction and validation of the DPM

The steps for the construction and validation of DPM were as follows by “survival” and “glmnet” package in R: (1) screen for DEIGRC associated with patient prognosis by univariate Cox regression; (2) reduce the number of genes to solve the multicollinearity problem by least absolute shrinkage and selection operator (LASSO) analysis; (3) construct DPM by multi-factor Cox regression; (4) calculate risk scores for patients with COAD in the UCSC Xena, GSE17536, and GSE39582 datasets; (5) demonstrate the relationship between risk scores and patient prognosis using Kaplan-Meier (KM) survival curves; (6) compare the predictive efficacy of prognostic models with that of constructing prognostic model genes using receiver operating characteristic (ROC) curves and concordance index (C-index).

### Construction of nomogram

To verify that DPM was a risk factor independent of the patient's clinical traits, we performed multiple bioinformatics algorithms on risk scores and clinical traits in that order. The C-index was then applied to compare predictive efficacy. Finally, the DPM and clinical features nomogram was produced using the “rms” package in R.

### Comparing differences in clinical characteristics between subgroups

The clinical data from the downloaded UCSC Xena database were compiled, and those lacking certain clinical features were eliminated. The distribution and differences in clinical traits between subgroups were visualized.

### Comprehensive analysis of immunological profiles between subgroups

The “CIBERSORTx” package in R was used to calculate the proportions of 22 immune cell types in patients with COAD and to analyze the differences in the proportions of immune cells between subgroups. The best “cutoff” values for different immune cell proportions were then obtained using the surv_cutpoint and surv_categorize functions of the “survminer” package in R. The patients were categorized into subgroups with high proportions based on these values. The optimal “cutoff” values for different immune cell ratios were obtained by using the surv_cutpoint and surv_categorize functions in the “survminer” package, based on which the patients were categorized into high- and low-proportions subgroups, and the survival differences between the groups were compared by means of the Kaplan-Meier (KM) survival curve. In addition, the immune cell function scores of COAD patients were estimated with the help of the R software packages “GAVA” and “GSEABase”, and patients were categorized into high- and low-scoring subgroups based on the optimal “cutoff” values, and the survival differences between the subgroups were further evaluated. Finally, the immunomarker expression differences were compared between the subgroups.

### Comparison of differences in immunotherapy and targeted therapy between subgroups

Tumor immune dysfunction and rejection (TIDE) scores can be utilized to predict the efficacy of immunotherapy. High TIDE scores predict high immune evasion potential, indicating that patients with tumors may not be suitable for immunotherapy. Transcriptomic data from COAD patients were uploaded to the TIDE database to calculate patients' T-cell-related scores. Compare the predictive efficacy of DPM, TIDE, and tumor inflammatory signature (TIS) models via ROC curves.

### Bioinformatics analysis of molecular mechanisms for the development of liver metastases in COAD

Regarding metastasis, the liver is one of the most susceptible organs in COAD patients, and liver metastasis is one of the main causes of the high mortality rate among COAD patients. In this work, we sought to forecast the occurrence of liver metastases in COAD patients using the DPM. However, we could not do so with adequate accuracy. The GSE6988 dataset was employed to explore mechanisms of liver metastases in COAD. We screened DEGs in COAD samples with liver metastases and COAD samples without liver metastases. Build protein-protein interaction (PPI) network and find core genes through the GeneMANIA database and Cytoscape software. Perform GO and KEGG enrichment analysis of DEGs by R.

### Acquisition of clinical tumor tissue

The First Affiliated Hospital of Hebei North University provided the COAD tissues and adjacent noncancerous tissue for this research. The tissues were collected within 30 minutes of the surgical specimens being separated. Connective and fatty tissue were removed on the edges of the fresh surgical specimens. Before being covered in RNA protective solution and kept at -80° C, the tissue surface was immediately cleaned of blood and grime with pre-cooled PBS solution or normal saline.

### RT-qPCR

A sterile, enzyme-free EP tube with 300 μl of Solution R1 was filled with about 30 mg of tissue, which was then ground for 1-2 min. Centrifuge at 13,000 rpm for 15 seconds once there were no longer any visible tissue fragments. The supernatant was transferred to a new EP tube and mixed thoroughly with 500 μl of Solution R2. Centrifuge at 13,000 rpm for 30 seconds after adding the mixed liquid to the adsorption column to remove the waste product. Add 500 μl of RNA Wash Buffer to the adsorbent column, centrifuge at 13,000 rpm for 15 seconds, and repeat once. The cDNA Reverse Transcription Kit's instructions were followed to build the reverse transcription system, and reverse transcription was performed at 37° C for 15 min and then at 85° C for 5 seconds. Set up the reaction schedule after preparing the amplification system following the amplification kit's instructions. Cyclic reaction: 95° C, 10 s, then 60° C, 30 s, 40 cycles; pre-denaturation: 95° C, 30 s, 1 cycle.

### Availability of data and material

The data sets used and/or analyzed during the current study are available from the corresponding author upon reasonable request.

## RESULTS

### Download results of IRGs and transcriptome datasets

**Figure 1:**
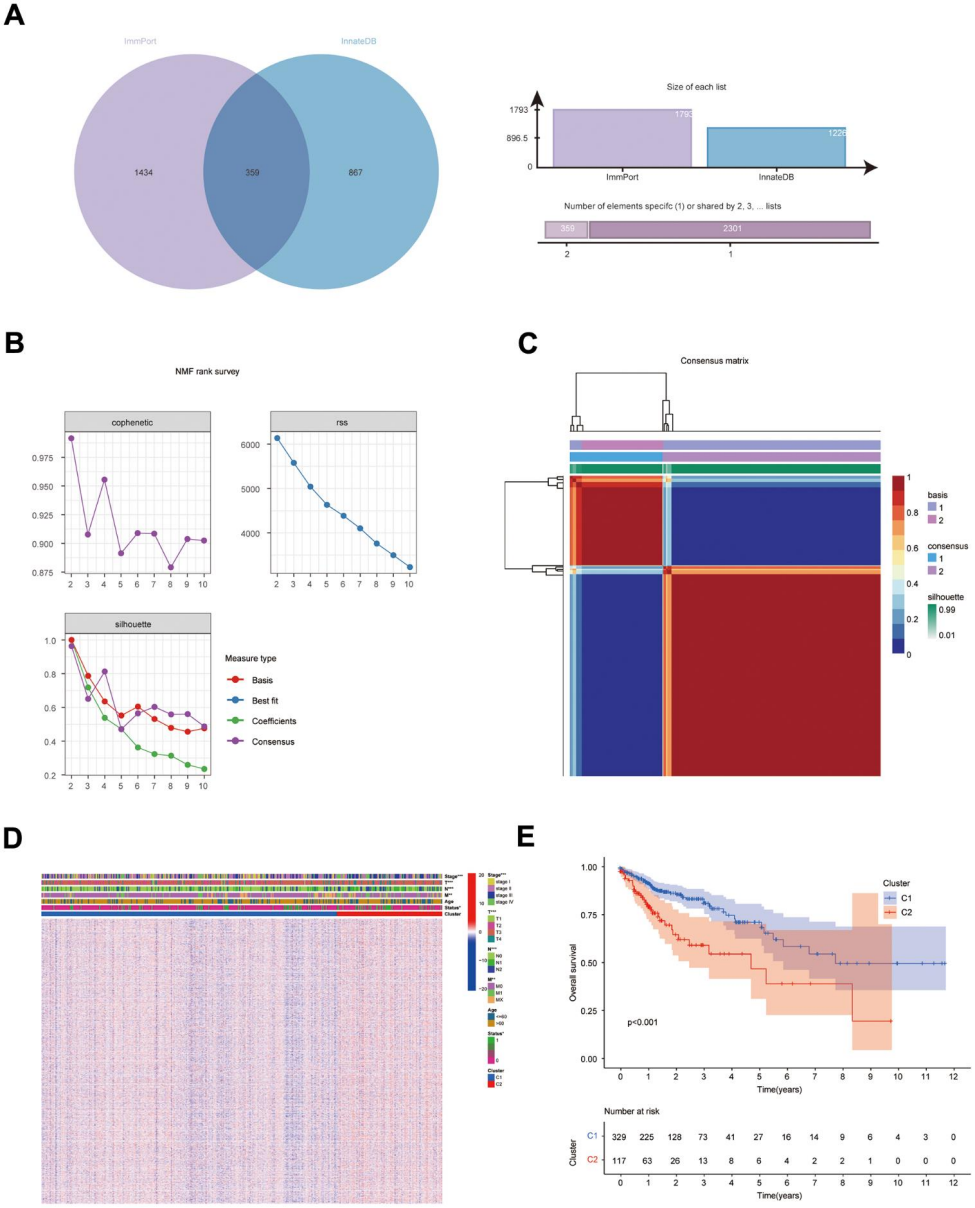
**Results of NMF clustering analysis.** (**A**) Venn diagram of IRGs in ImmPort database and InnateDB database; (**B**) Rss, dispersion, and cophenetic plots of the number of screened NMF clusters; (**C**) Consensus diagram of NMF clustering of IRGs; (**D**) Distribution of IRGs and clinical traits in immune subgroups; (**E**) KM survival curves for immune subgroups.

1793 and 1226 IRGs were obtained from the ImmPort database (Supplementary Table 1) and InnateDB database (Supplementary Table 2), respectively, and 2660 IRGs were obtained after merging (Figure 1A). We obtained sequencing data from 349 normal samples and 471 COAD tissue samples from the UCSC Xena database and identified DEGs using this dataset. Furthermore, we acquired two COAD datasets, GSE17536 and GSE39582, from the GEO database, consisting of chip data for 177 and 573 COAD samples, respectively. These datasets were utilized to validate the prognostic impact of the DEIGRC Prognostic Model (DPM) across different datasets. Additionally, we curated the GSE109211 dataset from the GEO database, encompassing chip data for 67 tumor samples subjected to targeted therapy. This dataset served to authenticate the accuracy and efficacy of our developed DPM in forecasting clinical benefits of targeted therapy.

### Identification of immune subtypes in COAD

The NFM technique was employed to cluster the expression data of IRG from the COAD sample (UCSC Xena database). Cluster consensus maps from 2 to 10 (Supplementary Figure 1), rss maps, dispersion maps, and cophenetic maps (Figure 1B) were used to determine the optimal number of clusters 2 (Figure 1C), which resulted in the division of patients into cluster 1 (C1) and cluster 2 subgroups (C2). Heat maps were applied to display the expression and clinical traits of IRGs in the subgroups (Figure 1D). The KM survival analysis revealed that subgroup C1 had a better prognosis (Figure 1E).

### Detection of important gene modules in subtypes

**Figure 2:**
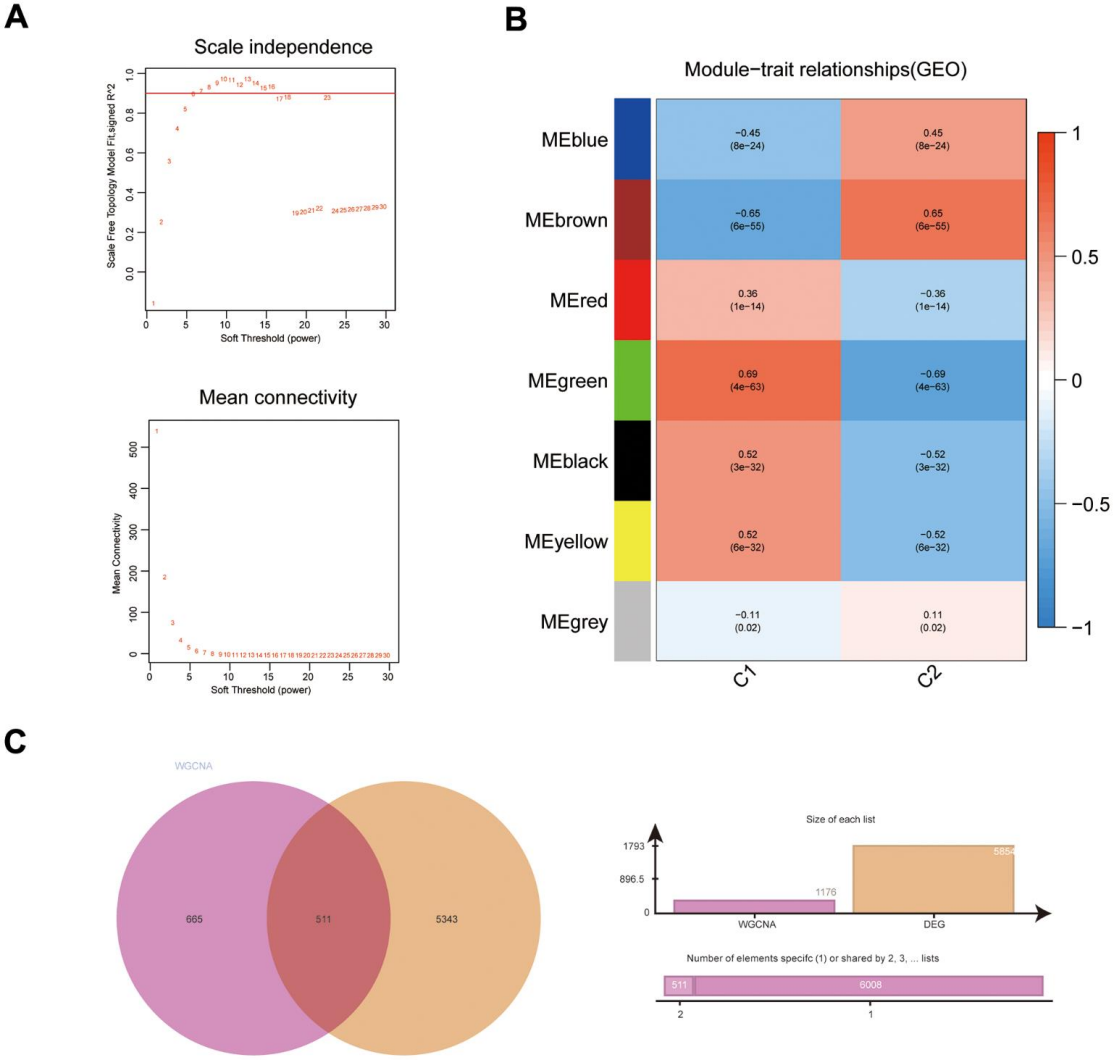
**WGCNA results for immune subgroups.** (**A**) Results of screening for soft threshold power; (**B**) correlation analysis of gene modules with immune subgroups; (**C**) Venn diagram of gene modules with high correlation to immunophenotyping and DEGs.

The DEGs (C1 *vs.* C2) obtained with the top 5000 *p*-values were then used for WGCNA. The “sft” function in R helped to obtain the best soft threshold 7 measurements (Figure 2A). Seven gene modules were created by calculating correlations between gene modules and subgroups and merging substantially identical gene modules (Figure 2B). With correlation coefficients above 0.6, the brown and green gene modules had the strongest connection with immunophenotyping. Volcano and heat maps demonstrate DEGs (normal colon tissues *vs.* COAD tissues) in the COAD patients from UCSC Xena database (Supplementary Figure 2A, 2B). Intersecting genes of gene modules with the highest subgroup correlation (green and brown) and DEGs (COAD) were defined as DEIGRC (Figure 2C).

### Functional and pathway enrichment analysis of DEIGRC

**Figure 3:**
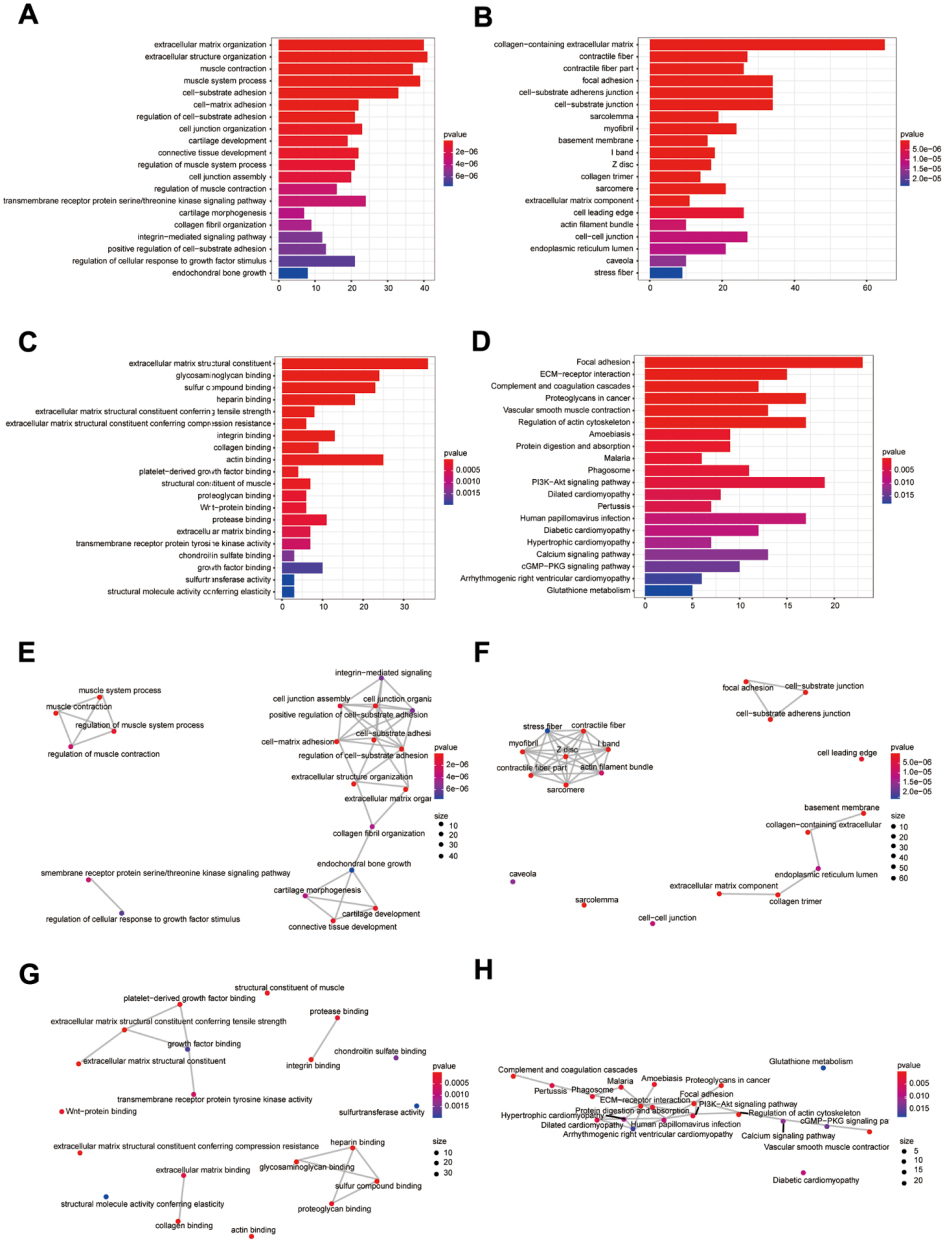
**Bioinformatics analysis of DEIGRC.** (**A**–**D**) Bar charts of BP, CC, MF, and KEGG enrichment analysis results; (**E**–**H**) Correlation analysis of the results from BP, CC, MF, and KEGG enrichment analysis.

Figure 3A–3D illustrates the enrichment results for GO and KEGG. Results of KEGG enrichment analysis demonstrated that DEIGRC was mainly enriched in the Calcium signaling pathway, Glutathione signaling pathway, cGMP-PKG signaling pathway, etc.

### Construction and validation of DPM

**Figure 4:**
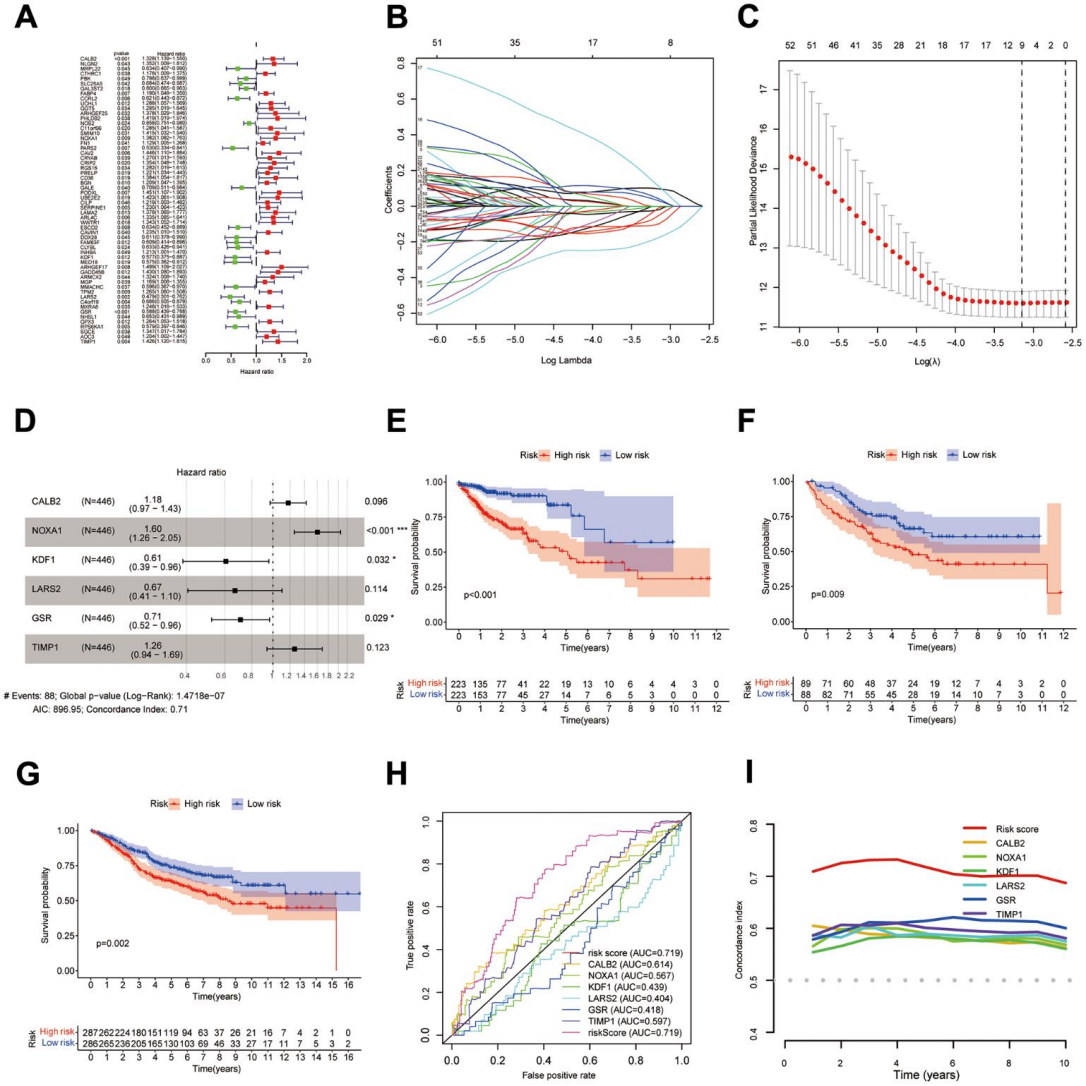
**Construction of DPM.** (**A**–**D**) Results of univariate Cox, LASSO, and multivariate Cox analyses of DEIGRC; (**E**–**G**) KM survival curves for the COAD patients from UCSC Xena database, GSE17536 and GSE39582 datasets; (**H**, **I**) ROC curves and C-indexes compare predictive efficacy of DPM with those of the genes used to construct the model.

58 DEIGRC associated with prognosis in COAD patients were obtained by univariate Cox regression analysis (Figure 4A, *p*<0.05). LASSO and multi-factor Cox regression analyses were performed sequentially on the 58 DEIGRC, resulting in the construction of the DPM consisting of 6 genes (Figure 4B–4D). CALB2, NOXA1, and TIMP1 were positively related to great poor prognosis, whereas KDF1, LARS2, and GSR were positively related to excellent prognosis (Supplementary Figure 3A–3F). In the COAD patients from UCSC Xena database, GSE17536 and GSE39582 datasets, the DEIGRC model displayed a strong ability to predict prognosis (Figure 4E–4G). The DEIGRC model had a greater prediction performance than the genes used to build it, according to the ROC curve and the C-index (Figure 4H, 4I).

### Construction of nomogram

**Figure 5:**
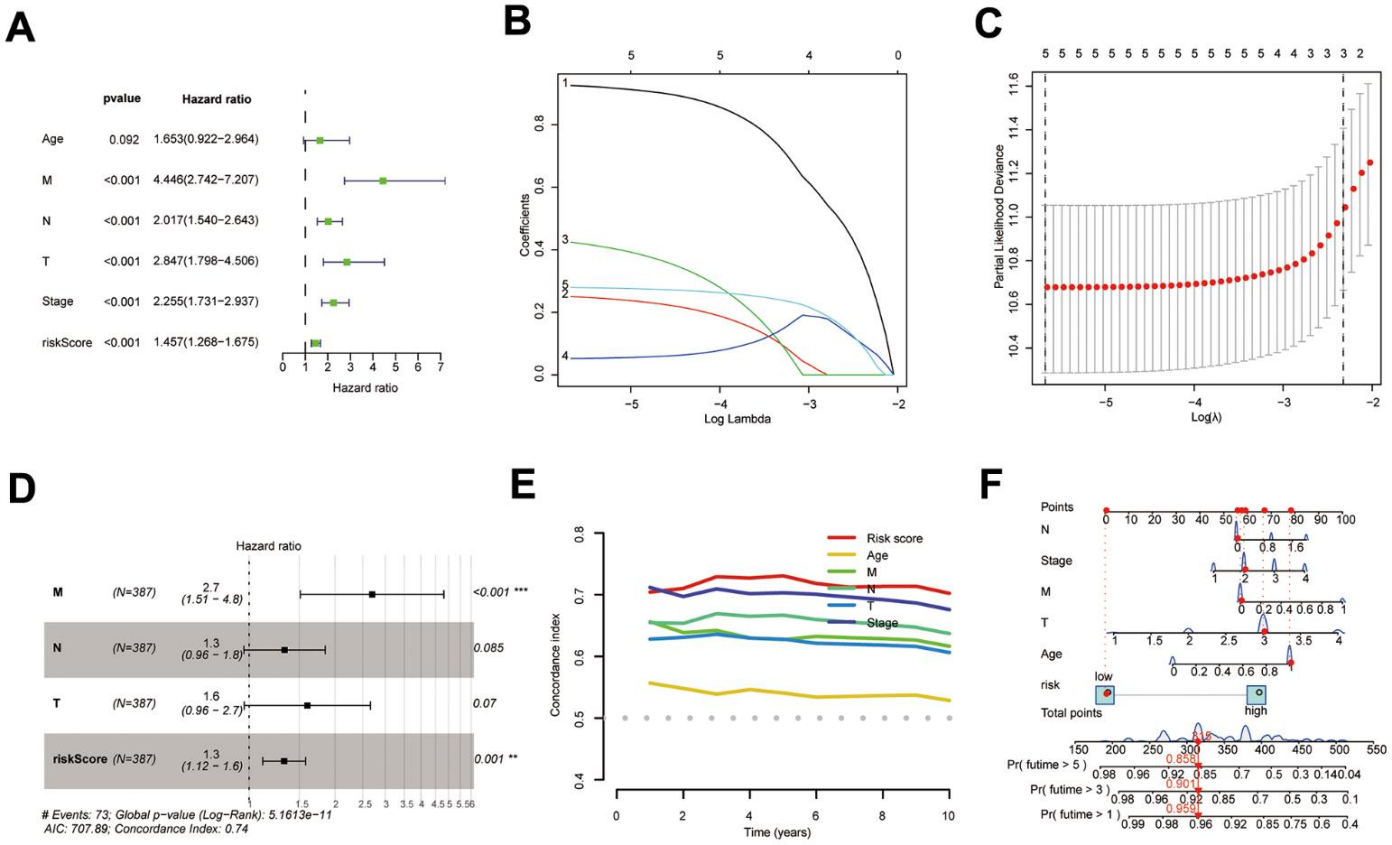
**Construction of the COAD nomogram.** (**A**–**D**) Results of univariate Cox, LASSO, and multi-factor Cox analyses of prognostic models and clinical traits; (**E**) C-index comparing the predictive efficacy of the prognostic model with that of clinical traits; (**F**) The COAD nomogram was constructed using prognostic models and clinical traits.

The results of KM survival curves indicated that Age was not associated with patient prognosis (Supplementary Figure 4A, *P*=0.089), while tumor (T), metastasis (M), node (N), and Stage were associated with patient prognosis (Supplementary Figure 4B–4E, *p*<0.001). DPM and clinical traits were sequentially analyzed by bioinformatics algorithms, and results revealed that DPM could be used as a prognostic indicator independent of clinical traits (Figure 5A–5D). Results of the C-index demonstrated that risk score had better predictive efficacy than clinical features in COAD patients (Figure 5E). DPM and clinical traits were further used to construct a nomogram for forecasting prognosis (Figure 5F).

### Clinical characteristics of different subgroups of patients

**Figure 6:**
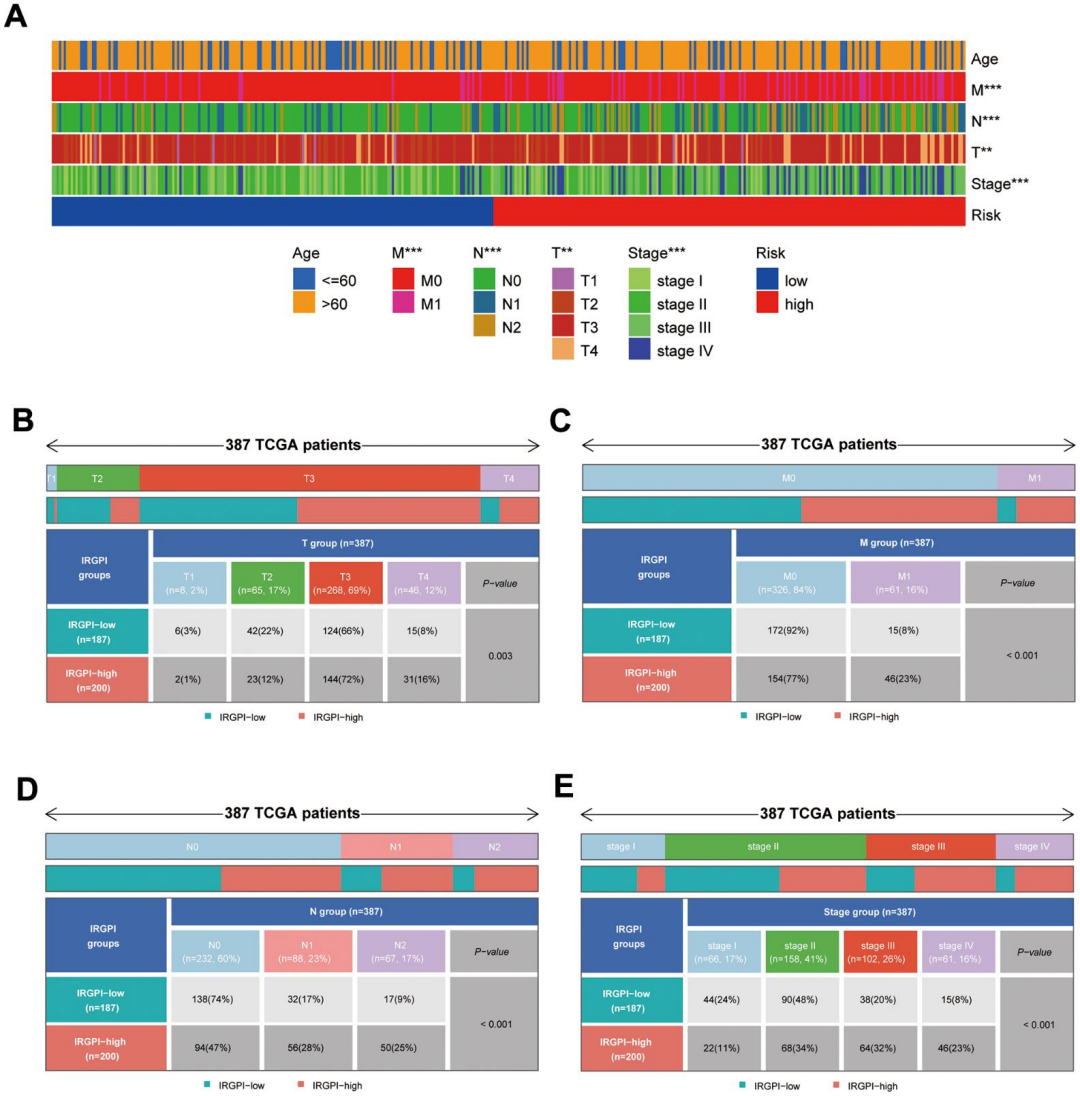
**Results of differential analysis of clinical traits between subgroups.** (**A**) Distribution of clinical traits between subgroups; (**B**–**E**) Results of differential analysis of T-stage, M-stage, N-stage, and stage between subgroups.

After collecting and filtering the clinical information of COAD patients from the UCSC Xena database, heat maps were created to show the clinical traits of the subgroups. The distribution of T, M, N, and Stage between subgroups was substantially different (Figure 6A, *p*<0.01). Using the “ComplexHeatmap” package in R, it is possible to more clearly demonstrate the distribution of T, M, N, and Stage between subgroups (Figure 6B–6E).

### Immunologic characteristics and therapeutic treatments of patients between different subgroups

**Figure 7:**
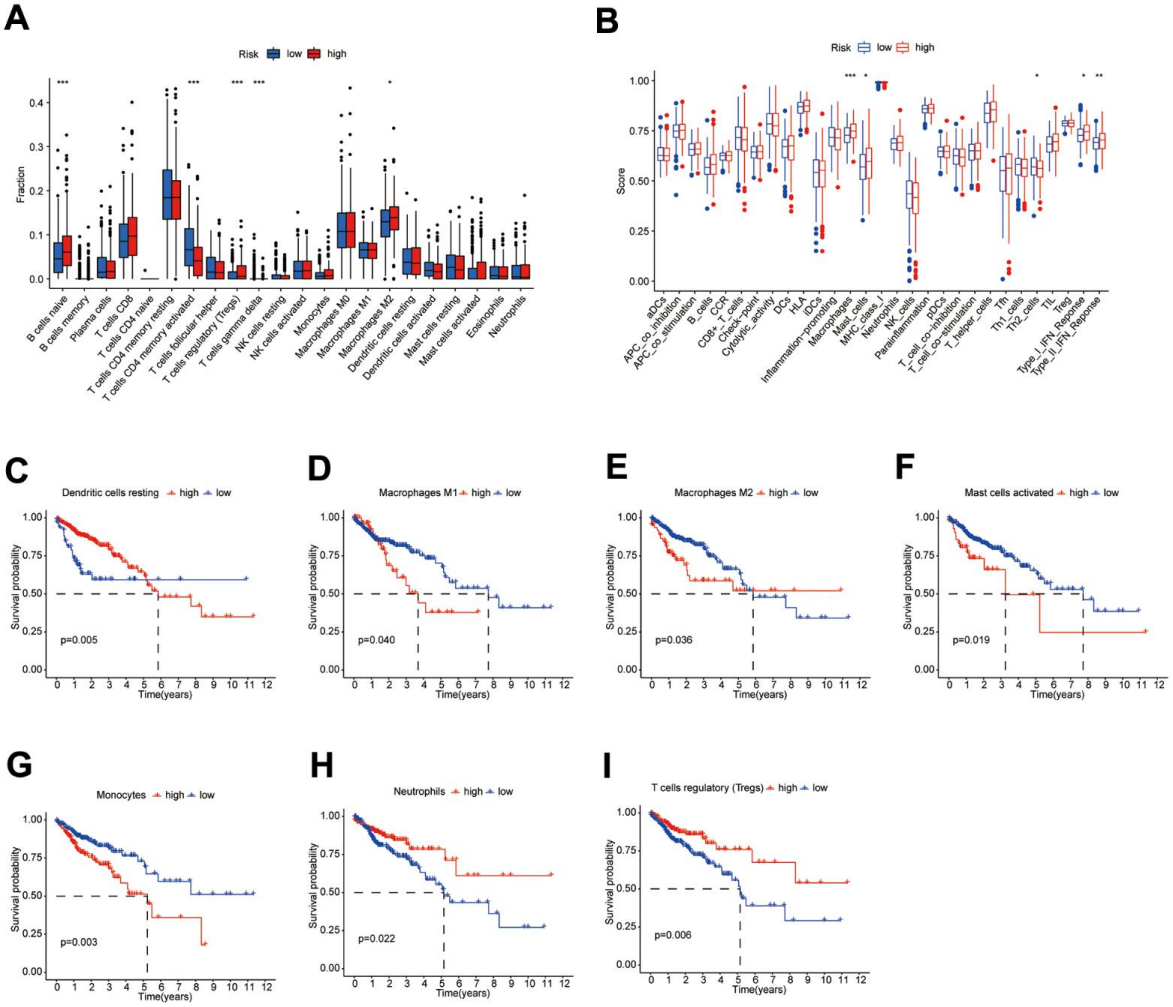
**Differences in immune characteristics between subgroups.** (**A**, **B**) Differences in the proportion of immune cells and Immune cell function scores between high- and low-risk subgroups; (**C**–**I**) KM survival curves for the proportion of 7 immune cells in high- and low-risk subgroups.

**Figure 8:**
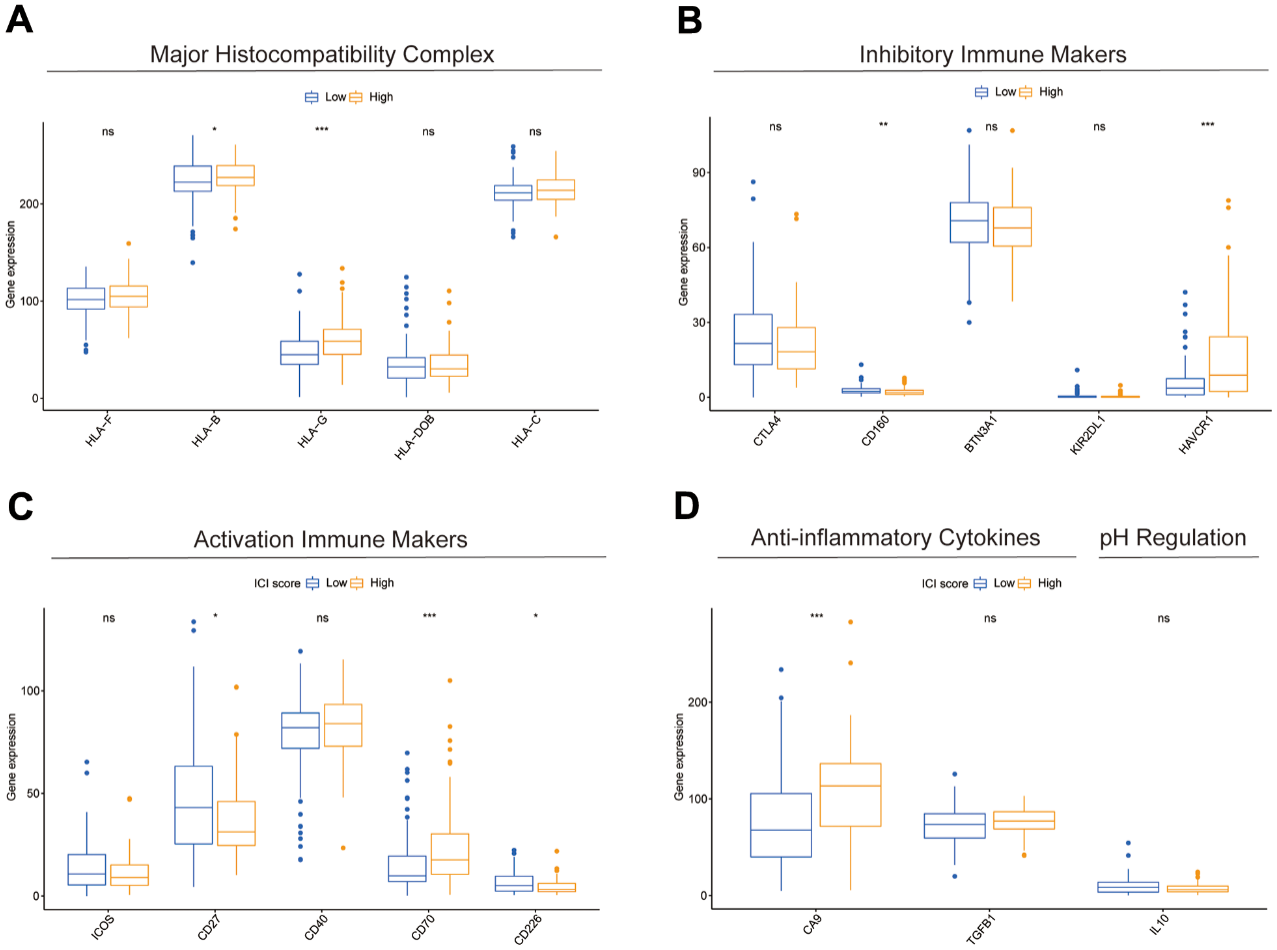
**Differences in immune-related markers between subgroups.** (**A**–**D**) Differences in expression of multiple types of immune marker genes between subgroups.

Results of the 22 immune cell type ratios are displayed in Supplementary Figure 5A. The immune cell proportions and immune cell function scores for the various subgroups are shown in Figure 7A, 7B. The relationship between immune cell ratios and the prognosis of COAD patients was demonstrated through KM survival curves (Figure 7C–7I). The prognosis of high-score and low-score subgroups was connected with functional scores of aDCs, APC co-inhibition, APC co-stimulation, and other immune cell function (Supplementary Figure 5B–5P). According to box plots of immunological marker expression, HLA-B, HLA-C, HLA-F, CD70, and TGFB1 were strongly upregulated and CD160, HAVCR-1, and ICOS were strongly downregulated in the high-risk subgroup (Figure 8A–8D).

**Figure 9:**
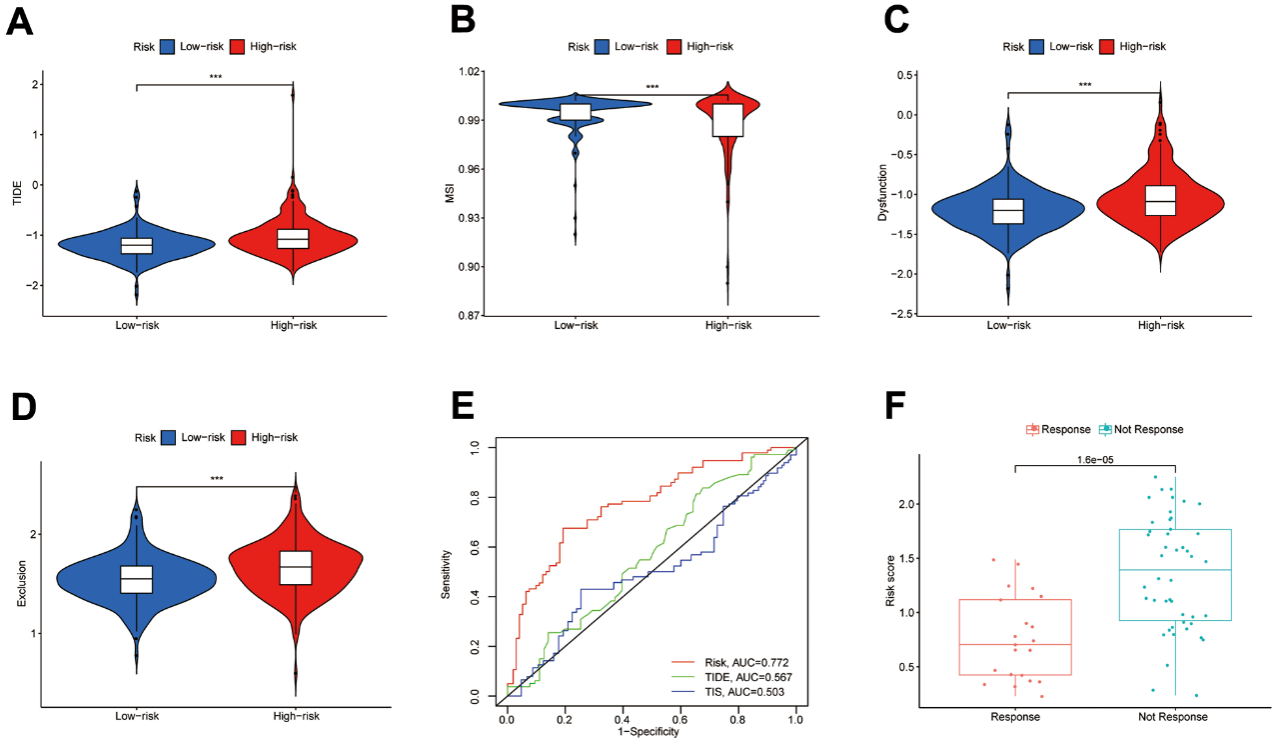
**Value of the DPM in predicting immunotherapy outcomes.** (**A**–**D**) Differences in TIDE, MSI, and T-cell dysfunction and exclusion scores between subgroups; (**E**) ROC curves comparing prognostic efficacy of prognostic models with those of TIDE and TIS; (**F**) Differences in risk scores between immune responsive and non-responsive patients (GSE109211 dataset).

Low-risk subgroup had larger microsatellite instability (MSI) scores, while the high-risk subgroup had larger TIDE, T-cell rejection, and T-cell dysfunction scores (Figure 9A–9D). We, therefore, considered that the DEIGRC model may be used to forecast which patients might benefit from immunotherapy. ROC curve results showed DPM had higher predictive efficacy than the TIDE and TIS models (Figure 9E). Patients who responded to sorafenib had lower risk scores than those who did not, so the DEIGRC model might be able to be used to predict the effectiveness of sorafenib treatment (Figure 9F).

### Potential mechanisms for the development of liver metastases from COAD

**Figure 10:**
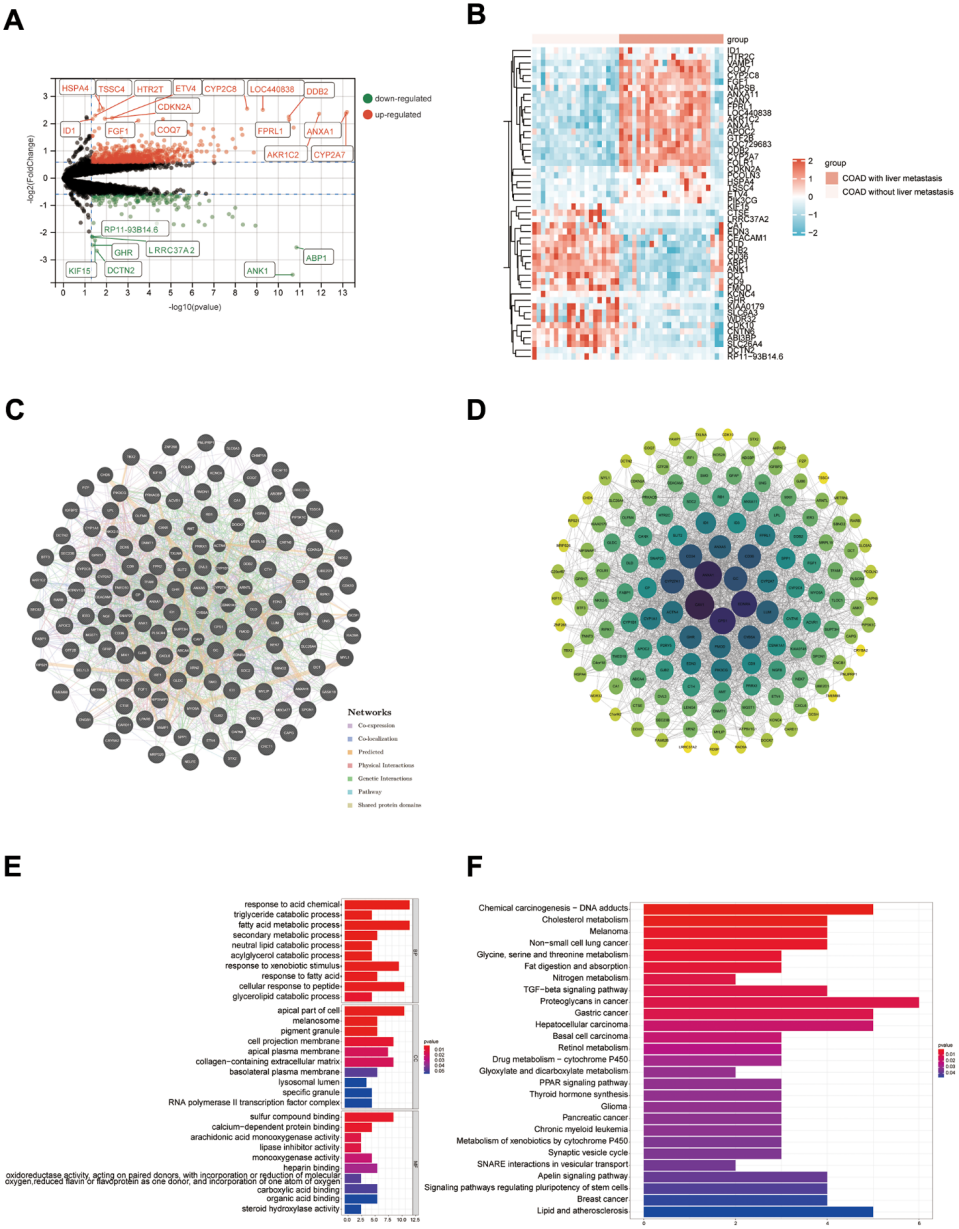
**Results of bioinformatics analysis of potential mechanisms for developing liver metastasis in COAD.** (**A**) Volcano map; (**B**) Heat map (only the top 25 highly and lowly expressed genes are shown); (**C**, **D**) PPI networks were constructed from the GeneMANIA database and Cytoscape software, respectively; (**E**, **F**) Results of GO and KEGG enrichment analysis, respectively.

DEGs in COAD samples with liver metastasis and COAD samples without liver metastasis were presented in Figure 10A. The heat map demonstrated expression distribution for the 25 most highly expressed genes and the 25 most lowly expressed genes in COAD with liver metastases samples (Figure 10B). The DEGs were applied to construct a PPI network, and core genes were further screened based on the degree of nodes using Cytoscape software (Figure 10C, 10D). The main genes contributing to the development of liver metastases in COAD patients include CAV1, ANXA1, CPS1, EDNRA, and GC. Figure 10E, 10F show the findings of the GO and KEGG enrichment analyses, respectively. According to KEGG enrichment analysis, potential mechanisms underlying the development of liver metastases in COAD patients may be Fat digestion and absorption, Proteoglycans in cancer and Nitrogen metabolism.

### Expression levels of 6 DPM genes in clinical tissue samples

**Figure 11:**
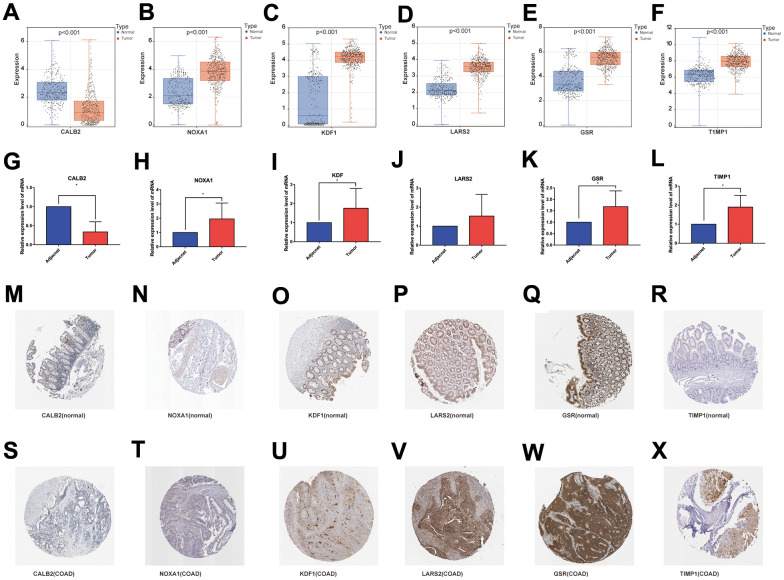
(**A**–**F**) mRNA expression levels of the 6 genes modeled in the databases (TCGA and GTEx); (**G**–**L**) RT-qPCR results of the 6 genes modeled in the collected clinical samples; (**M**–**R**) and (**S**–**X**) immunohistochemical results of the 6 genes modeled in normal colon tissue and COAD tissue.

This study obtained surgical samples from 10 pairs of colon cancer patients. Using bar graphs to depict the individual mRNA expression from each of the 6 genes in the COAD dataset (TCGA and GTEx), we discovered that CALB2 was only lowly expressed in COAD tissues, whereas the other 5 genes were highly expressed (Figure 11A–11F). The mRNA expression of these 6 genes was then further investigated by reverse transcription quantitative polymerase chain reaction (RT-qPCR) in the collected adjacent and COAD tissues (Table 1), and the outcomes were found to agree with the gene expression in database (Figure 11G–11L).

**Table 1:** Primer sequences for the 6 genes of DMP.

**Primer name**	**Specific sequence**	**Length**
CALB2	Forward 5’-TTCCATCCACCACCTTGCCAATG-3’	24
	Reverse 5’-AAAGGAGCACGCCGAGTAAAGAAG-3’	23
NOXA1	Forward 5’-CCGCCAGGCTGTGCTTCAAC-3’	22
	Reverse 5’-TGGTCACGGCTTGGTCAAATGC-3’	20
KDF1	Forward 5’-CAGCAGCATCACGCAGGACTAC-3’	21
	Reverse 5’-CAGCAGCCCGAGTTGAACGAC-3’	22
LARS2	Forward 5’-CTACACCATCAGCGACACCATAGC-3’	22
	Reverse 5’-GCGGCATTTTCAGCAGGCAATC-3’	24
GSR	Forward 5’-CTGGAGTGCGGTGGTGCTATTTC-3’	23
	Reverse 5’-ATGGTGGTGCGTGCCTGTAATTC-3’	23
TIMP1	Forward 5’-ATCCTGTTGTTGCTGTGGCTGATAG-3’	24
	Reverse5’-CGCTGGTATAAGGTGGTCTGGTTG-3’	25

The Human Protein Atlas database (https://www.proteinatlas.org/) was used to retrieve the expression of the 6 genes at the protein level in healthy colon tissues and colon cancer tissues, and the results were consistent with RT-qPCR (Figure 11M–11X).

## DISCUSSION

COAD accounts for approximately 70% of patients with CRC [31]. The highly heterogeneous features of COAD make its clinical treatment more complex and difficult. Immunotherapy, as one of the most likely therapeutic cures for tumors, is classified into two main categories: immune cell therapy and immune checkpoint therapy, and has been applied in the clinical treatment of a variety of tumors with encouraging therapeutic results [32–35]. For treating COAD patients, the immune checkpoint-related medications nabolutumab and pablizumab are now being used in clinical trials [36]. Nevertheless, due to a lack of biomarkers and models to predict patient response and prognosis to immunotherapy, low response rates to immunotherapy, and the fact that only some patients indicate treatment, immunotherapy has experienced several challenges in clinical practice [37, 38]. IRG model has been built to evaluate the prognosis of patients with tumors based on transcriptome sequencing data [39, 40]. However, the potential mechanisms between IRGs, COAD, and immune features remain underdeveloped. In the current investigation, NFM and WGCNA algorithms were employed to filter DEIGRC, and prognostic models were constructed using one-factor Cox, LASSO, and multi-factor Cox algorithms. The DPM demonstrated superior predictive efficacy as a prognostic factor independent of clinical features and could be employed for predicting clinical outcomes, according to a further bioinformatics investigation.

Using the NFM and WGCNA algorithms, we finally identified 511 DEIGRCs. The prognostic model constructed in this study consisted of 6 DEIGRCs, of which CALB2, NOXA1, and TIMP1 were risk factors for COAD patients, while KDF1, LARS2, and GSR were protective factors for COAD patients. GSE17536 and GSE39582 datasets also validated the model's prognostic value. A calcium receptor protein called CALB2 has the ability to bind Ca^2+^ [41, 42]. In colon cancer tissues, the expression level of CALB2 was significantly higher than that of normal colon epithelial cells, and at the same time, the expression level of CALB2 was positively correlated with the metastasis of local lymph nodes and other organs [43, 44]. Zhang et al. discovered that CALB2 up-regulated MMM9 and down-regulated E-cadherin, which in turn encouraged colon cancer cells to invade and migrate [45]. NoxA1, a homolog of p67^phox^, is thought to be an activator of Nox1 [46, 47]. Nox1 and its regulators NoxO1 and NoxA1 are expressed greater in human gastric and intestinal adenocarcinomas than in normal gastric mucosa, suggesting that Nox protein activation could be a sign of tumor transformation [48]. In COAD cells, NoxA1 is crucial for the functional and reactive oxygen species-dependent development of endocryptal fossas [49]. TIMP1, as a soluble protein, is a member of the tissue inhibitor family of metalloproteinases [50]. TIMP1 is secreted by cancer cells, fibroblasts, and endometrial cells, and has been associated with a poor prognosis in a variety of malignancies [51–53]. TIMP1 is regarded as a novel predictive biomarker for colon cancer due to its close relationships to processes and functions pertaining to the metastasis, proliferation, and apoptosis of cancer cells [54]. TIMP1 is also thought to be a viable target for the treatment of colon cancer since it was shown that it stimulates the growth and invasiveness of right-sided colon cancer cells via the FAK/Akt signaling pathway [55]. By encoding a precursor to mitochondrial leucine-tRNA synthetase, amino-tRNA synthetase LARS2 regulates the translation of mitochondria-encoded genes [56]. Breast cancer tumor growth and proliferative capacity were increased in mouse mammary glands with single allele LARS gene deletion [57]. LARS2-secreting B-cell subsets are highly correlated with the prognosis of CRC patients and promote immune escape of colorectal cells [58]. KDF1 is a crucial regulator of epidermal differentiation and an inhibitor of cell proliferation [59]. For tissue homeostasis and cancer prevention, KDF1 plays a crucial function in preserving the right balance between cell division and differentiation. Reduced KDF1 expression has been discovered in cancer cells, and it has been demonstrated to correlate with patient survival positively and negatively correlate with tumor grade [60]. Chromosome 8p12, where GSR is located, is frequently deleted in CRC [61]. There is growing evidence that the deletion of chromosome 8p lowers the survival rates of cancer patients and enhances the aggressiveness and metastatic potential of CRC [62–64]. Glutathione peroxidase utilizes the reducing capacity of GSR to scavenge excess reactive oxygen species in the cytoplasm, thereby preventing oxidative stress-driven cancer progression [65]. The mechanism of COAD cell differentiation and proliferation is intimately linked to GSR [66].

We discovered statistically significant variations in the proportion of B cells naive, T cells regulatory, Macrophages M2, and T cells CD4 memory activated between subgroups. B cells play an important role in the tumor microenvironment. By secreting cytokines, B cells naive can prevent lung cancer cells from proliferating, and the presence of B cells naive is favorably correlated with a positive prognosis for lung cancer patients [67]. Through cell interaction and bodily fluids, T cells govern various immune cells, including macrophages [68]. Eliminating T cell regulation increases T cells’ capacity to attack tumor cells and boosts the patient’s immunological response to tumors [69, 70]. Macrophages are part of the immune system and contain M0 macrophages, M1 macrophages, and M2 macrophages, with MO macrophages polarized into M1 or M2 types. M1 macrophages can boost the inflammatory response and destroy tumor cells, while M2 macrophages have the efficacy to suppress the inflammatory response and promote tumor cell proliferation and metastasis [71]. T cells CD4 memory activated was positively correlated with good prognosis in breast and bladder cancer [72, 73].

KM survival curve indicated low-risk subgroup had a better prognosis. The TIEDE database, which was created to score the T-cell function of the samples by computing the transcriptome sequencing data of the samples, could be applied to forecast the immunotherapeutic outcome of patients [74]. In the high-risk subgroup, TIDE, T-cell Dysfunction and Exclusion scores were higher than in the low-risk subgroup. The higher these scores, the greater the likelihood of immunological escape and the worse the patient’s immunotherapeutic outcome. Additionally, the ROC curve’s predictions of patient survival time were more accurate than those of the TIDE and TIS models. Additionally, since the DEIGRC model only includes 6 genes, it is easier for clinical prediction in COAD patients.

## CONCLUSIONS

We constructed DPM consisting of an immune-related model that can predict the prognosis for COAD patients. Further studies revealed that this model may be used to predict the suitability of immunotherapy and targeted therapy for oncology patients, which may help in the clinical management of oncology patients. We also identified potential molecular mechanisms and therapeutic targets for developing liver metastases in COAD patients, which may contribute to fundamental research related to liver metastases in COAD and the development of related new drugs.

## Supplementary Material

Supplementary Figures

Supplementary Table 1

Supplementary Table 2
