# Supplementary material for: Investigating gene signatures associated with immunity in colon adenocarcinoma to predict the immunotherapy effectiveness using NFM and WGCNA algorithms
Source: Aging (Albany NY). 2024 May 13;16(9):7596–621. doi: 10.18632/aging.205763 (PMC11131999; doi:10.18632/aging.205763)
Supplement: Supplementary Figures [file aging-16-205763-s001.pdf]

SUPPLEMENTARY FIGURES

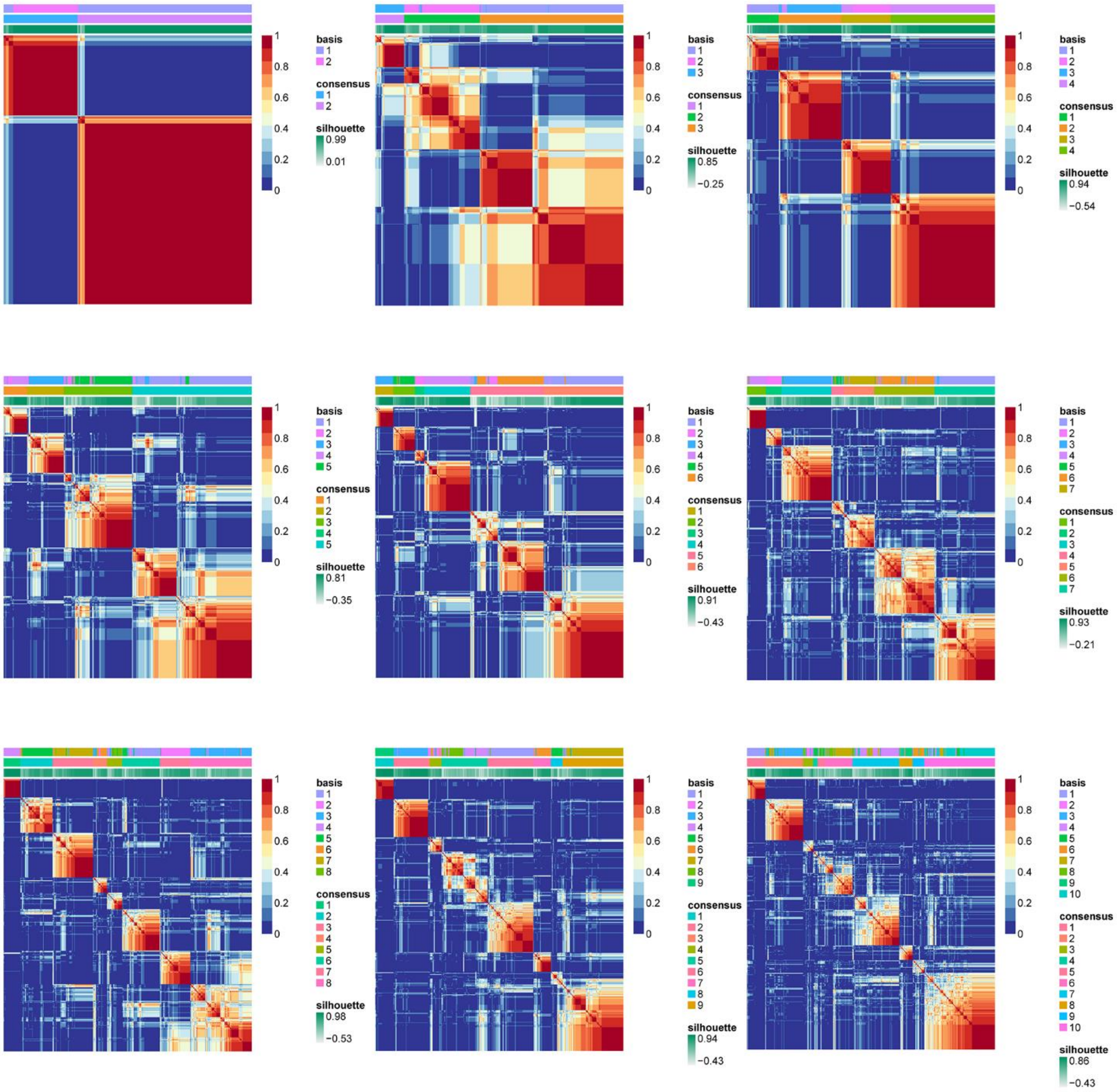

Supplementary Figure 1. Results of NFM analysis for classification numbers from 2 to 10.

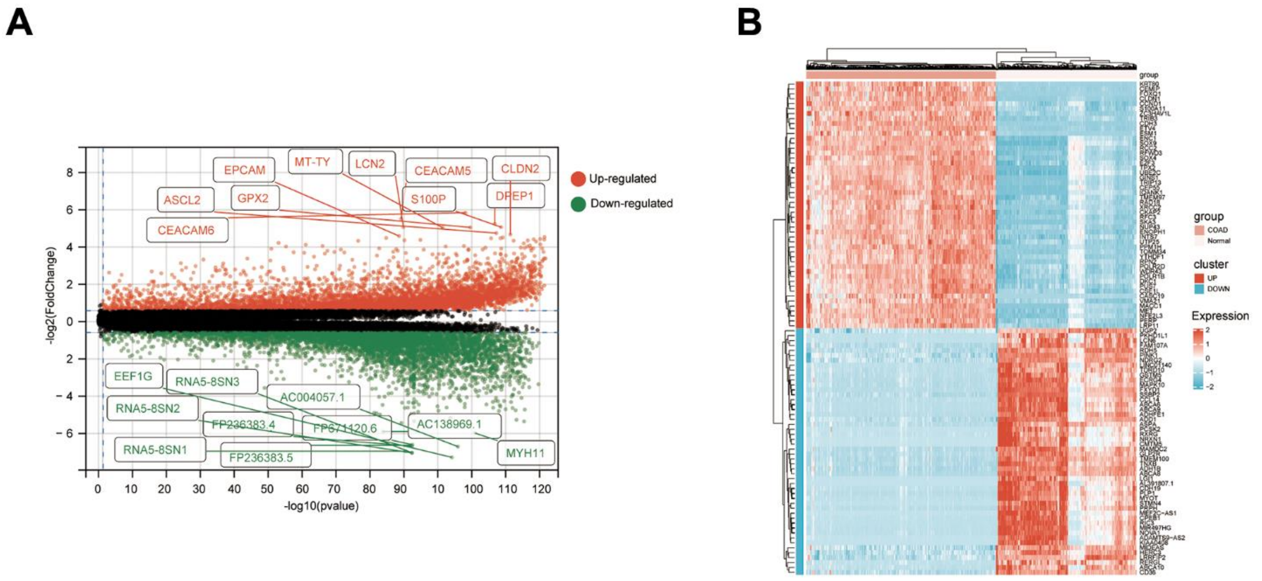

Supplementary Figure 2. DEGs in COAD patients. (**A**) Volcano plot; (**B**) Heat map (only the top 50 highly and lowly expressed genes are shown).

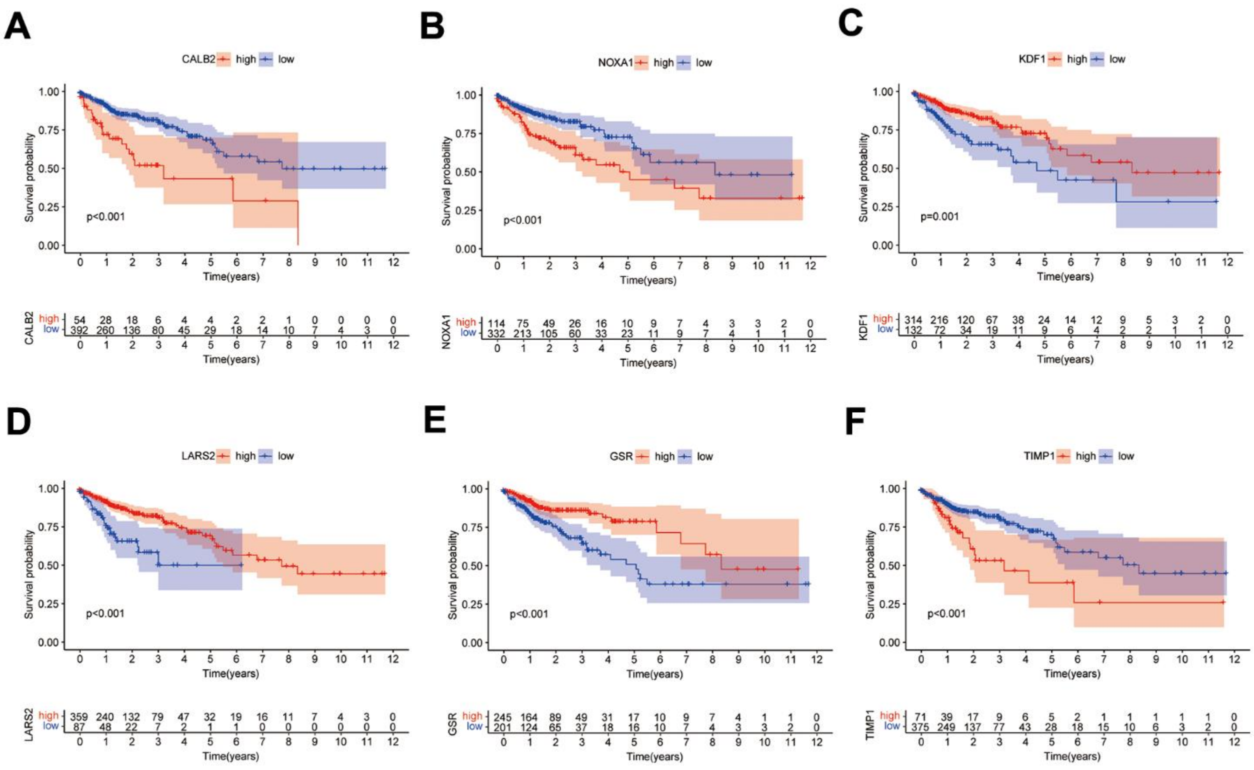

Supplementary Figure 3. KM curves for genes used in the construction of DPM. (**A**–**F**) KM survival curves for CALB2, NOXA1, KDF1, LARS2, GSR, and TIMP1.

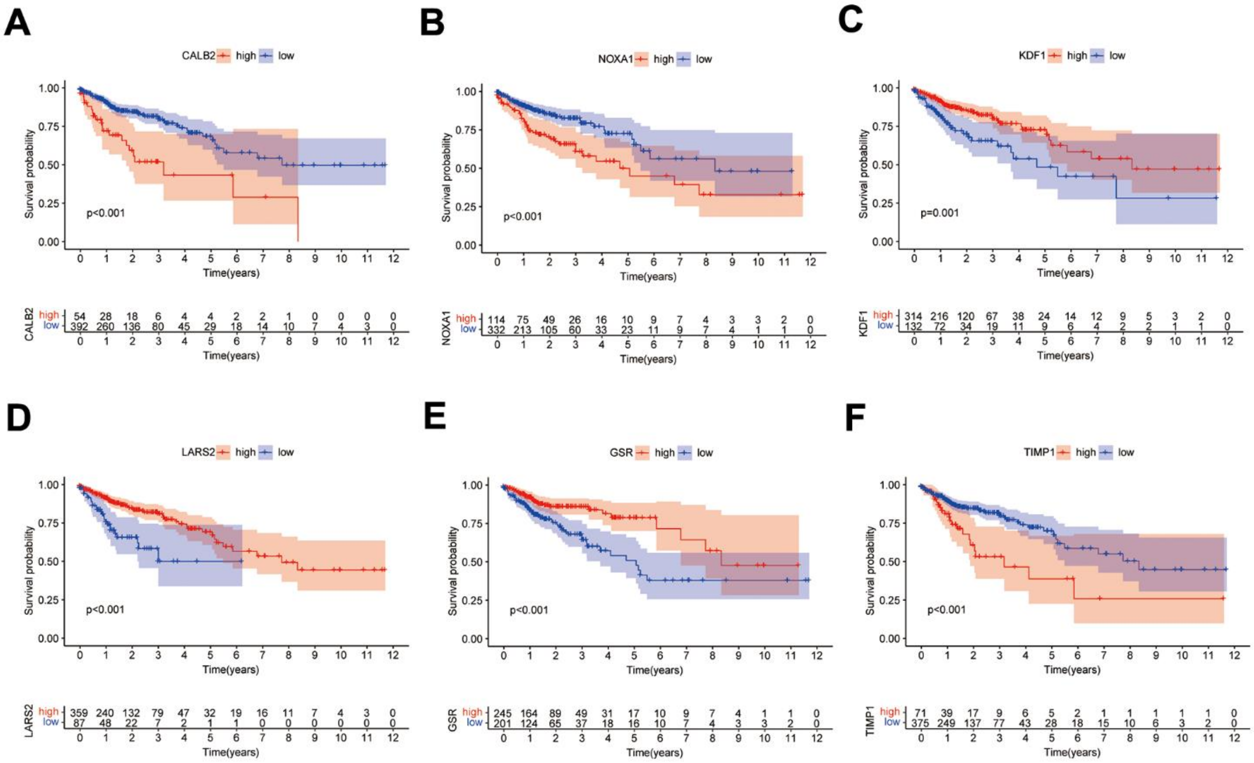

Supplementary Figure 4. Relationship between clinical traits and prognosis of patients with COAD. (**A**–**E**) KM survival curves for Age, T-stage, M-stage, N-stage, and stage.

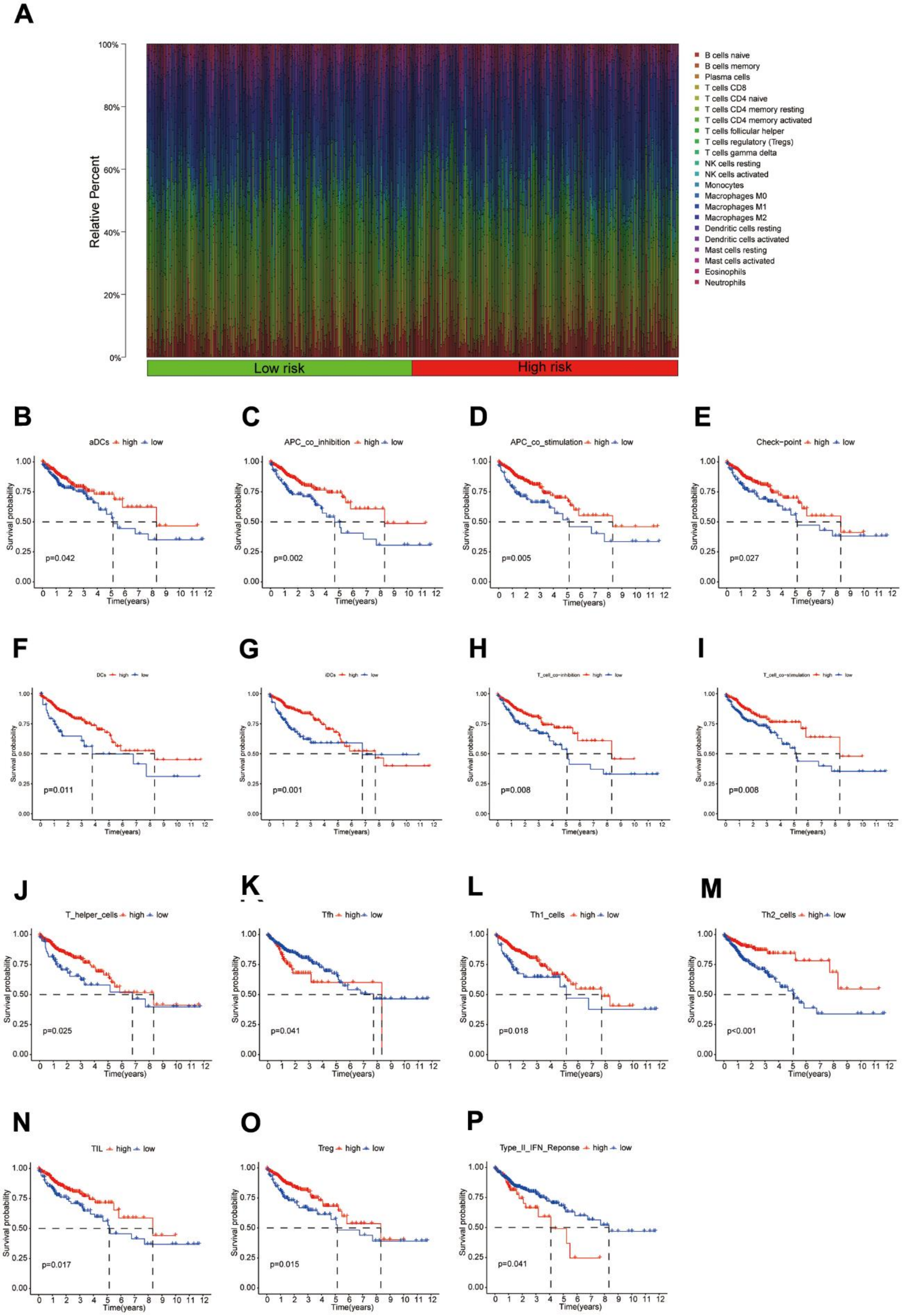

Supplementary Figure 5. Relationship between immune cell function scores and the prognosis of patients with COAD. (**A**) The proportion of immune cell distribution between subgroups; (**B**–**P**) KM survival curves for 15 immune cell function scores significantly associated with prognosis in COAD patients ($p<0.05$).
